# “Trustworthiness,” confidence in estimated effects, and confidently translating research into clinical practice

**DOI:** 10.1186/s40945-023-00162-9

**Published:** 2023-04-06

**Authors:** Sean P. Riley, Brian T. Swanson, Chad E. Cook

**Affiliations:** 1 Department of Rehabilitation Sciences, University of Hartford, West Hartford, CT USA; 2 Duke Center for Excellence in Manual and Manipulative Therapy, Duke University, Durham, NC USA; 3 Doctor of Physical Therapy Program, University of Hartford, 200 Bloomfield Ave, West Hartford, CT 06117 USA; 4 Doctor of Physical Therapy (DPT) Division, Duke University, Durham, NC USA; 5 Department of Orthopaedics, Duke University, Durham, NC USA

**Keywords:** Bias, Confidence intervals, Evidence-based practice, Prospective studies, Registries, Systematic review

## Abstract

*Trustworthy*, preprocessed sources of evidence, such as systematic reviews and clinical practice guidelines, are crucial for practicing clinicians. Confidence in estimated effects is related to how different the outcome data were between the two groups. Factors including the effect size, variability of the effect, research integrity, research methods, and selected outcome measures impact confidence in the estimated effect. The current evidence suggests that post-randomization biases cannot be ruled out with a high degree of certainty in published research, limiting the utility of preprocessed sources for clinicians. Research should be prospectively registered to improve this situation, and fidelity with prospective intent should be verified to minimize biases and strengthen confidence in estimated effects. Otherwise, discussions related to preprocessed literature, including *P*-values, point estimates of effect, confidence intervals, post-randomization biases, external and internal validity measures, and the confidence in estimated effects required to translate research into practice confidently, are all moot points.

## Background

Trustworthiness is Essential. The American Physical Therapy Association recognizes that it is essential to establish “*quality*” by generating, validating, and disseminating research evidence [1]. Because most clinicians fail to acquire, are unable, or are uninterested in the skills necessary for critically appraising the research evidence [2] - which is required for evidence-based practice - trustworthy, high-quality, preprocessed sources of evidence are crucial for practicing clinicians. Unfortunately, research evidence exists on a spectrum, including publications of questionable trustworthiness. This viewpoint outlines challenges related to trustworthy evidence and educates readers on what may improve confidence in research findings.

## How has trustworthiness been traditionally identified?

It is *assumed* that published research summaries that critically appraise and synthesize research evidence, such as systematic reviews (SRs) and clinical practice guidelines (CPGs), are trustworthy [2]. Historical elements of trustworthiness have traditionally been established by identifying between-group differences based on a) *P* values, b) estimates of effect, and c) precision [3, 4]. *P* values determine if there is a statistically significant difference between compared groups. In contrast, point estimates of effect (How large is the between-group difference?) are used to determine if the difference is meaningful by being larger than the minimum clinically important difference (MCID) (a clinically important difference from the patient’s perspective). Finally, the precision of the estimated effect (How large is the confidence interval?) is used to determine if the observed effect was accurate. Ideally, the confidence interval (CI) should be small, as large CIs hinder the likelihood that repeated findings will be similar and the between-group differences, as it suggests that what was observed could be a chance finding. This historical perspective regarding trustworthy, clinically meaningful between-group differences is based on a *big* assumption. The *big* assumption is that the researchers established the research question(s) and methods before collecting data, followed these methods, and the data were analyzed and interpreted based on the original research questions and methods (compliance and transparency). Additionally, it is assumed that what occurred after data collection and initial analyses were performed involved high fidelity with the protocol and appropriate blinding. Very recently, there has been an evolving story on how research trustworthiness needs to be established. The recommended tools that are presently used to assess the quality of reporting and study validity may not be valid unless there is established fidelity of the published manuscript with an established research record.

## How has trustworthiness in research evidence evolved? (See Table 1)

**Table 1:** How has trustworthiness in research evidence evolved?

	**Year**	**Obstacle**	**Source**
*P* Values	2016	The *P* value is arbitrary and does not convey any evidence related to the effect size.	Yaddanapudi LN. The American Statistical Association statement on P-values explained. J Anaesthesiol Clin Pharmacol. 2016;32(4):421-3.
Estimates of Effect and Precision	2021	Assumptions are that the effect size and the variability of the effect are sufficient to attain confidence in estimating the treatment effect.	Elkins MR, Pinto RZ, Verhagen A, Grygorowicz M, Soderlund A, Guemann M, et al. Statistical inference through estimation: recommendations from the International Society of Physiotherapy Journal Editors. J Man Manip Ther. 2022;30(3):133-8.
Post-Randomization Biases in Research	2021	Post-randomization biases occur after data collection, and initial results are obtained.	Andrade C. HARKing, Cherry-Picking, P-Hacking, Fishing Expeditions, and Data Dredging and Mining as Questionable Research Practices. J Clin Psychiatry. 2021;82(1).
Post-Randomization Biases in RCTs	2021	Post-randomization biases cannot be ruled out in randomized clinical trials (RCTs) in International Society of Physiotherapy Journal Editors (ISPJE) member journals 64.5% of the time.	Riley SP, Swanson BT, Shaffer SM, Sawyer SF, Cleland JA. The Unknown Prevalence of Post-randomization Bias in 15 Physical Therapy Journals: A Methods Review. J Orthop Sports Phys Ther. 2021;51(11):542-50.
Post-Randomization Biases in SRs	2022	Post-randomization biases cannot be ruled out in RCTs within systematic reviews (SRs) in ISPJE member journals 8.9% of the time; 58.3% of the SRs included RCTs with unknown external validity; and 35% of the RCTs included in the SRs were low in methodological quality.	Riley SP, Swanson BT, Shaffer SM, Somma MJ, Flowers DW, Sawyer SF. Is the quality of systematic reviews influenced by prospective registration: a methods review of systematic musculoskeletal physical therapy reviews. J Man Manip Ther. 2022:1-14.

### Problems with *P* values

In 2016, the American Statistical Association developed a statement on *P* values [3]. One of the six essential principles outlined, recognizing that a *P* value is arbitrary and does not convey any evidence related to the effect size in randomized clinical trials (RCTs), is particularly relevant when discussing confidence in estimated effects [3]. It is well known that the *P* value is not enough to establish that the difference is meaningful; point estimates of effect and their confidence intervals better serve this purpose. A statistically significant difference may be related to a type 1 statistical error, which occurs when a difference is identified when in reality, none exists –common in large, overpowered sample sizes. These errors can be identified as statistically significant differences that are smaller than the measurement error of the outcome measures used or a difference that is smaller than the clinically meaningful differerence from the patients perspective (MCID).

### Estimates of effect and precision

In 2021, the International Society of Physiotherapy Journal Editors (ISPJE) published guidance that statistical significance testing should be abandoned and that statistical inference through estimation should be used [4]. The ISPJE suggests that the estimated observed effect should be as large as the MCID of the outcome measures used. Additionally, the ISPJE indicated that the confidence intervals (CI) should be used to ensure that the observed effect was not a potentially meaningless chance finding [4]. Their primary assumptions are that the effect size and the variability of the effect are sufficient to attain confidence in estimating the treatment effect. An additional challenge is that the baseline values of patient-reported outcome measures have been shown to influence the MCID value and accuracy, as does the method used in calculating the MCID, suggesting that the MCID may not help interpret if the findings are clinically meaningful from the patient’s perspective [5].

### Problems with post-randomization biases

In 2021, it was comprehensively recognized that confidence in the estimated treatment effect could be impacted by post-randomization biases that occur after data collection and initial results are obtained. Examples include: HARKing (generating a hypothesis after the results are known); Cherry-picking (selectively reporting and discussing data that supports a hypothesis), p-hacking (running statistical analyses until statistical significance is found), and data dredging or data mining (looking for relationships between variables just because the data are available) [6].

A 2021 methodological SR of RCTs involving musculoskeletal physical therapy interventions published in ISPJE member journals demonstrated that it *could not be* determined if researchers followed their original research question, followed their methods, or reported their study consistent with the established research record 64.5% (89/138) of the time [7]. This was secondary to the RCTs being retrospectively registered, unregistered, or having unclear registrations. Furthermore, of the ISPJE member journals that required prospective clinical trial registration, it was established that 8.2% (4/49) of the RCTs changed their primary research question, and 16.3% (8/49) changed their primary outcome measures [7]. In addition, four of these questionable publications were rated as having Physiotherapy Evidence Database (PEDro) scores of 7-8/10 [7]. This suggests that, without verifying if an RCT was published consistent with its prospective intent, these questionable studies would likely be synthesized in preprocessed evidence with inappropriately high confidence in estimated effect.

In 2022, a methodological SR on musculoskeletal physical therapy interventions in an ISPJE member journal explored the prospective registration of SRs and the RCTs that were used to create them [8]. This study found that verified prospectively intent could not be established to rule out post-randomization biases in 95% (19/20) of the identified SRs and in 91.1% (154/169) of the RCTs that were synthesized to create these SRs [8]. This suggests that many SRs related to musculoskeletal interventions appearing in ISPJE member journals have unknown validity. Additionally, 35% of the RCTs included in the SRs in this study were fair to poor in methodological quality, with PEDro scores of less than six out of ten [8]. These findings suggest that post-randomization bias cannot be ruled out in a large proportion of SRs and the RCTs that are used to create them. Therefore, recommended tools used to establish research validity may not be valid if this criterion cannot be established first. This is important when translating research into practice recommendations using the Grading of Recommendations, Assessment, Development and Evaluations (GRADE) approach, as confidence in the estimated effect is established through moderate to high-quality evidence [9]. If fidelity with prospective clinical trial registration cannot be determined, RCTs have an unclear risk of bias, as any tool used to establish RCT quality cannot help since we are unsure if they were conducted as planned [7]. This is a significant threat to the trustworthiness of our highest levels of preprocessed research evidence used by clinicians in clinical practice.

## Conclusion

How can we create trustworthy preprocessed research? Minimizing the possibility of a chance finding means that preprocessed sources (e.g., SRs and CPGs) must be synthesized based on high-quality research that produces high confidence in the reported point estimated effects. Establishing trust in research is related to research integrity (verified prospective registration), appropriate methodology (external and internal validity), and selecting meaningful and relevant outcomes from the patient’s perspective [10]. The GRADE approach might help in embracing the uncertainty of the evidence; however, the evidence’s certainty might be affected if the primary literature’s prospective intent cannot be established first.

Confirmation and post-randomization biases cannot be ruled out with a high degree of certainty in the ISPJE member journals related to musculoskeletal interventions. Although it has been recognized that confidence in estimated effects is essential for confidently translating clinical research into practice [9], research is lacking on which variables must be considered, prioritized, and are most important when making this determination.

If post-randomization biases cannot be ruled out, it threatens the confidence in the validity of the reported research and trustworthiness. Further, consistency must be secured between the registered research question, primary outcomes, analysis appropriateness, and results interpretation. If the integrity of the RCTs comprising our preprocessed literature is not improved, the ongoing discussions regarding *P* values, point estimates of effect, confidence intervals, and the confidence in the point estimate of the effect required to translate research into practice confidently are effectively moot points. Ultimately, expecting the practice clinician readers to navigate through the murky waters of quality appraisal is unrealistic if the certainty of the evidence cannot be established.

## Data Availability

Not applicable.

## Abbreviations

CPGs: Clinical Practice Guidelines
CI: Confidence Interval
GRADE: Grading of Recommendations, Assessment, Development and Evaluations
ISPJE: International Society of Physiotherapy Journal Editors
MCID: Minimum Clinically Important Difference
PEDro: Physiotherapy Evidence Database
RCTs: Randomized Clinical Trials
SRs: Systematic Reviews

## References

[1] Association APT. Guiding principles to achieve the vision 2019. Available from: https://www.apta.org/siteassets/pdfs/policies/guiding-principles-to-achieve-vision.pdf. Accessed 22 Feb 2023.

[2] Tikkinen KAO, Guyatt GH (2021). Understanding of research results, evidence summaries and their applicability-not critical appraisal-are core skills of medical curriculum. BMJ Evid Based Med.

[3] Yaddanapudi LN (2016). The American Statistical Association statement on P-values explained. J Anaesthesiol Clin Pharmacol.

[4] Elkins MR, Pinto RZ, Verhagen A, Grygorowicz M, Soderlund A, Guemann M (2022). Statistical inference through estimation: recommendations from the International Society of Physiotherapy Journal Editors. J Man Manip Ther.

[5] Boyer CW, Lee IE, Tenan MS (2022). All MCIDs are wrong, but some may be useful. J Orthop Sports Phys Ther.

[6] Andrade C (2021). HARKing, cherry-picking, p-hacking, fishing expeditions, and data dredging and mining as questionable research practices. J Clin Psychiatry.

[7] Riley SP, Swanson BT, Shaffer SM, Sawyer SF, Cleland JA (2021). The unknown prevalence of postrandomization bias in 15 physical therapy journals: a methods review. J Orthop Sports Phys Ther.

[8] Riley SP, Swanson BT, Shaffer SM, Somma MJ, Flowers DW, Sawyer SF. Is the quality of systematic reviews influenced by prospective registration: a methods review of systematic musculoskeletal physical therapy reviews. J Man Manip Ther. 2022:1-14. 10.1080/10669817.2022.2110419. 10.1080/10669817.2022.2110419 PMC10288892 35942578

[9] Andrews JC, Schunemann HJ, Oxman AD, Pottie K, Meerpohl JJ, Coello PA (2013). GRADE guidelines: 15. Going from evidence to recommendation-determinants of a recommendation’s direction and strength. J Clin Epidemiol.

[10] Chow M, Birdwell J. Confidence in research: researchers in the spotlight. Elsevier; 2022. Available from: https://policycommons.net/artifacts/3159336/confidence_in_research-full_report/3957225/.

